# Confronting trauma as a team: therapists’ experience with providing intensive trauma-focused treatment, within a framework of therapist rotation

**DOI:** 10.1186/s12913-026-14497-z

**Published:** 2026-04-14

**Authors:** Kristin Stotesbury, Hilde Einarsdatter Danielsen, Anders Lillevik Thorsen, Ottar Ness, Marius Veseth

**Affiliations:** 1 Department of Clinical and Biological Psychology, University of Bergen, Bergen, Norway; 2 Kronstad District Psychiatric Center, Haukeland University Hospital, Bergen, Norway; 3 Department of Psychology, Norwegian University of Science and Technology, Trondheim, Norway; 4 Bergen Center for Brain Plasticity, Haukeland University Hospital, Bergen, Norway; 5 Centre for Crisis Psychology, University of Bergen, Bergen, Norway; 6 Department of Education and Lifelong Learning, Norwegian University of Science and Technology, Trondheim, Norway; 7 Department of Psychiatry, District General Hospital of Førde, Førde, Norway

**Keywords:** Intensive trauma-focused treatment, Therapist rotation, Mental health care systems, Qualitative study

## Abstract

**Background:**

Therapists report that working in a framework of therapist rotation decreases their fear of providing trauma-focused treatment, lessens avoidance behaviour and reduces therapists` drift from treatment protocol. In the present study we ask: How do therapists experience providing intensive, trauma-focused treatment within a framework of therapist rotation at Prompt Mental Health Care?

**Methods:**

Two focus group interviews were conducted with the team of therapists. Seven therapist meetings were observed, and field notes were made. Data was analysed using Reflexive Thematic Analysis.

**Results:**

The analysis yielded three themes: (1) It can be uncomfortable, but we stick to it, (2) Knowing that your team supports you, and (3) We need to treat people with trauma closer to home.

**Conclusion:**

This study demonstrates that teamwork can reduce barriers associated with providing trauma-focused treatment (TFT). It also shows that working in a functional team, within a framework of therapist rotation, has the potential to minimize therapist drift. The shared responsibility and support within the team makes it more feasible for therapists to provide TFTs. Consequently, a collaborative approach can enhance access to evidence-based treatments.

**Supplementary Information:**

The online version contains supplementary material available at 10.1186/s12913-026-14497-z.

## Background

Recent wars, pandemics, terrorist attacks and natural disasters have shown how entire populations have to deal with traumatic events (TE) and their aftermath. In addition to the kinds of adversities that affects large groups, people may also experience TEs on an individual level, such as sexual abuse, mugging, psychical abuse and mental abuse. Studies have shown that 70% of the world’s population will experience a TE during their life [1], experiences that have the existential power to shake us to the core of our being [2]. While many stay resilient after TEs, others can develop post-traumatic stress disorder (PTSD). Posttraumatic stress disorder (PTSD) is diagnosed based on persistent trauma-related symptoms, including intrusions and hyperarousal, resulting in functional impairment [3]. As well as PTSD, exposure to TEs is also associated with other adverse mental and physical health outcomes [4]. Considering the substantial burden imposed by traumatic events, effective ways to prevent and treat trauma should be a public global health priority [5, 6].

Today there is a range of different documented treatments for trauma. Together with trauma-focused cognitive behavioural therapy (TF-CBT), Eye Movement Desensitization and Reprocessing (EMDR) is included in PTSD treatment guidelines for PTSD [7]. A recent meta-analysis of randomised controlled trials showed that interventions both with and without a trauma focus are effective and acceptable in the treatment of PTSD [8]. As both treatments are effective it is continuously debated whether a trauma focus is necessary for an effective treatment of PTSD. Other studies have demonstrated heterogeneity in treatment effects [9–11] which might suggest that different patients benefit from different trauma treatments, implying that there is a need for a range of therapies.

Despite promising results for trauma-focused treatments, Hoppen et al. [8] showed that more patients discontinued TF-CBT compared to non-trauma-focused interventions. To prevent patients dropping out of trauma-focused treatment, intensive treatment programs for PTSD have emerged during the past 15 years with promising results [12–14]. As well as reducing rates of dropout, an intensive format accommodates a rapid recovery from symptoms of PTSD. A systematic review of intensive treatments for PTSD showed equivalent treatment outcomes to ordinary outpatient interventions, as well as reduced treatment dropout [15].

Restrictions during the COVID-19 pandemic challenged services to provide therapies over the internet. This was also the case for the service Prompt Mental Health Care, in this study. During the pandemic they provided EMDR online also referred to as iEMDR. When restrictions were lifted, the service in the present study continued carrying out this treatment online. The evidence of the effectiveness of online EMDR for PTSD is low, though there are some promising results [16–17]. Given the flexibility associated with online therapy, there is an urgent need to develop more knowledge about online EMDR for PTSD, and both qualitative and quantitative research are needed.

A Norwegian qualitative study investigated patients’ experiences with an intensive outpatient trauma treatment using EMDR and prolonged exposure (PE) [18]. The treatment was conducted over eight days and the therapist worked within a framework of therapist rotation. Therapist rotation means that a team of therapists treat a group of patients together and are equally responsible for them. For each individual therapy session the therapists rotate, hence patients meet all therapists who are part of the team during a treatment week [19–20]. The patients that took part in the study reported that the intensive treatment, within a framework of therapist rotation, promoted an effective and focused treatment that left less room for avoidance and safety-seeking behaviours [18].

Studies have shown that trauma-focused treatment is highly effective for PTSD [8, 21]. However, these treatments are underused amongst clinicians. A recent systematic review identified that the most dominant reported clinician barrier for providing evidence informed interventions for PTSD, was a lack of training [22]. The lack of training was linked to a lack of knowledge, an uncertainty of how to approach trauma and a lack of confidence in how to use evidence informed interventions [22] One proposed explanation is that limited training contributes to therapist-related fears, including concerns that patients’ symptoms may worsen as a result of the strong emotions elicited by trauma-focused work [22]. Such fears might feed into a belief of TFT’s being harmful, in contrast to effective [23–24]. Van Minnen et al. suggest that therapist rotation is a promising approach to improve implementation of trauma-focused treatment [20]. Therapists providing intensive trauma treatment, reported that working within a framework of therapist rotation decreased their fear of giving trauma-focused treatment, increased their perception of the patient’s readiness for trauma-focused treatment, lessened avoidance behaviour, and reduced them drifting away from treatment protocol [20]. The same study also found that many therapists were satisfied working within a therapist rotation model, and that the patients were able to establish a working alliance to the therapist team equal to a working alliance with an individual therapist [20]. Van Minnen et al. used a quantitative checklist to assess therapist experiences with therapist rotation, while our study provides a qualitative, in-depth exploration of these experiences in a primary-level community mental health service [20]. A recent study by Vaage-Kowalzik et al. examined therapists’ experiences with intensive, rotation-based trauma treatment in an inpatient setting, and found that therapist rotation reduced fear and increased adherence to trauma-focused protocols [25]. Our study complements this work by exploring similar questions in a primary-level, community-based service that delivers trauma-focused EMDR in a digital format. This extends knowledge about how team-based rotation models can be implemented in low-threshold, outpatient settings. Krampe et al. [26] suggested that therapist rotation has advantages for therapists, such as mutual support and shared responsibility. In the present study we ask: How do therapists experience providing intensive, trauma-focused treatment within a framework of therapist rotation at Prompt Mental Health Care?

## Methods

This qualitative study explores therapists’ experiences with providing Trauma-Focused Therapy (TFT) to patients seeking help at Prompt Mental Health Care. As described below, we used a reflexive thematic analysis [27] in our approach to the data which allowed us to also reflect on what we as researchers brought to the analysis.

### Study setting

In Norway, public mental health services are tax-financed and divided into three service levels. This study focused on the primary level of care and took place at a Community Mental Health Care Service in Norway, which incorporated a Prompt Mental Health Care team. Prompt Mental Health Care is free of charge and provides treatment to people above the age of 16. Although services vary across locations, they primarily offer treatment for mild or moderate mental health problems, such as mood disorders, anxiety disorders and sleep disorders. To our knowledge, there are now 75 such centres in Norway. No referrals are needed to access these services, and patients do not have to go through a diagnostic assessment before being given the right to treatment. These centres aim to provide patients with short term mental health care within one to two weeks of contacting the centre.

Therapists with different educational backgrounds work at these mental health care centres including clinical psychologists, nurses, social educators and social workers with education in psychotherapy, mainly cognitive therapy. Prompt Mental Health Care is inspired by the NHS Talking Therapies in the UK, known as Improving Access to Psychological Therapies [28]. NHS Talking Therapies was developed to improve access to evidence-based, NICE recommended psychological therapies [29].

### The treatment

The intensive TFT has a duration of five days. Before the treatment started, a treatment plan was formulated for each patient. Safety procedures included ensuring access to a private, undisturbed space, agreements regarding childcare, sick leave or external support, and development of individualized crisis plans when indicated. As part of the treatment plan patients and therapists developed a plan for physical activity and behavioral experiments to be carried out between sessions. The treatment framework was aligned with EMDR 2.0, emphasizing memory activation combined with substantial working-memory taxation [30]. The treatment week consists of 8 sessions of 90 min digital EMDR, referred to as iEMDR. After every session of iEMDR the therapists rotate, hence patients can meet up to 6 different therapists during the treatment week. Before and after every iEMDR session the therapists have a meeting where they talk about the sessions they have had, ensure adherence to the treatment protocol and plan the following sessions. In Table 1 these meetings are referred to as therapist meetings. The resource demands of the treatment involved six therapists who held individual sessions with patients conducted in parallel before rotating. Between four and five patients could take part each treatment week, ensuring that a therapist was always available for each patient.

**Table 1:** An overview of the treatment week with therapist rotation

Day 1	Day 2	Day 3	Day 4	Day 5
	Therapist meeting and rotation	Therapist meeting and rotation	Therapist meeting and rotation	Therapist meeting and rotation
Digital therapist and patient meeting: Introduction, information and psychoeducation	iEMDR	iEMDR	iEMDR	iEMDR
Therapist meeting	Therapist meeting and rotation	Therapist meeting and rotation	Therapist meeting and rotation	Therapist meeting
iEMDR	iEMDR	iEMDR	iEMDR	Digital therapist and patient meeting: Looking back and looking ahead
Therapist meeting	Therapist meeting	Therapist meeting	Therapist meeting	Therapist meeting

### Participants and recruitment

All team members of the Prompt Mental Health Care intensive trauma team (*N* = 6) were approached face-to-face by the first author and invited to participate in this study. They all accepted the invitation and provided signed informed consent prior to participation. The team consisted of six therapists, four women and two men between the age of 35 and 63 (M = 47). Four of them were Clinical Psychologists and two of them were Cognitive Therapists. One of the Cognitive Therapists was a Psychiatric Nurse, the other a Social Worker. Four had been part of the intensive trauma team since they started in the beginning of 2021, one had been part of the team for a year, and the sixth for three months. Four of the therapists had used EMDR for 7 years, one for two years and the sixth for three months.

This sample represents the entire team providing this specific intensive, team-based trauma-focused treatment, and thus constitutes a closed, homogenous group with shared, direct experience of the phenomenon under study. In line with Braun and Clarke’s [31–32] approach to reflexive thematic analysis, we frame our sample size and stopping decisions in terms of information power [33]. Consequently, the adequacy of the sample was assessed based on the richness and relevance of the data rather than on a fixed number of participants or the concept of saturation. We judged the data sufficient when the second round of multistage focus group interviews allowed us to deepen and refine the core themes identified in the initial analysis, and when the team’s feedback during member checking confirmed that these themes comprehensively captured the complexity of their shared experiences.

### Data collection

In this study, data was collected at the Prompt Mental Health Care service, where the therapists worked. Data collection included participatory observation [34] using written field notes [35] and two focus group interviews [36, 37]. No one else was present besides the participants and researchers when data was collected. The data material has only been used for this project and has not been used in any previous publications. The first author observed seven 60-minute therapist meetings during a therapy week. This allowed exploration of how the team worked together and how meetings were used. Field notes were taken whilst observing these therapist meetings, and more details were added immediately after each observation. Thereafter, the first and second authors conducted two focus group interviews with the therapist team, held two months apart in 2023. Four of the six participants attended the first focus group interview, two were absent due to illness. All six participants attended the second focus group interview. In these focus group interviews we used a participant-based interview method, multistage focus group interview [38], to explore therapist experiences. We chose this method so that we could include the participants in all stages of the research process. Participants’ input created ideas and influenced our decisions; in a sense they became co-researchers in the process of conducting this study. Several of the questions for the focus group interviews were developed in collaboration with the participants.

The interviews were conducted using a semi-structured interview guide. The first interview started with questions about providing trauma therapy at the Prompt Mental Health Care service. The interview then focused on therapist experiences with the therapy format which was intensive treatment over the internet, including both what was working well and what wasn’t. They were then asked questions about their experience working as a team. Other questions focused on therapist rotation. The interview ended with asking if there was anything they would like to change in the treatment program.

In multistage focus group interviews, follow-up interviews are typically structured around topics identified in the initial interview [38]. In the second interview we therefore decided to use quotes from the first interview alongside field notes from certain situations during the therapist meetings to initiate focused discussion. We did this to explore in more detail therapist experiences in specific situations. To illustrate, the following interaction recorded in field notes from one of the therapist meetings was presented to the group:

*Therapist 2: Yes*,* I felt I didn’t help her at all.*

*Terapist 1: Yeah, that’s not a good experience (…)*

*Therapist 2: I wouldn’t have been able to deal with this on my own. It’s really helpful to have someone else take over.*

A question was then asked: “What do you think when you hear this? Can you say something about what it’s like to be a team, in the face of this?” Interviews were conducted by the first and second author, audiotaped and thereafter transcribed verbatim by the interviewers. The interviews were both 83 min long. Transcripts were not returned to participants. However, we presented our identified themes to the therapist team to gather feedback on whether they accurately reflected their experiences.

**Table 2:** Overview of the qualitative research process

Focus Group 1	Initial Analysis	Focus Group 2	Final Analysis	Feedback to Participants
First interview	Preliminary analysis of first interview	Follow-up second interview	Comprehensive analysis of all data	Discussions and participants checking

### Data analysis

We used a reflexive thematic analysis [27, 31–32] to describe recurrent patterns across our data material. Thematic analysis was our choice due to its ability to facilitate thorough data exploration in a structured framework, as well as its strong emphasis on the researcher’s reflective engagement. We followed the six phases developed by Braun and Clarke [27] to guide our analysis:

1. The goal of this phase was to familiarise ourselves with the data. To align with the multistage focus group interview design, this process was conducted in two parts. Following the initial interview, KS, HED, and MV read through the material and discussed emerging themes as well as their own preconceptions and perspectives during a first analysis meeting. These discussions informed the focus and topics for the second interview. After the second interview, the familiarisation process was repeated. Formal coding of the material did not begin until both interviews and observations of the treatment week had been completed.
2. KS then coded the interviews and field notes based on our research question, using NVivo software to manage and organize the qualitative data. Codes captured descriptive elements of the data, in other words were made in a bottom-up process. An example from the coding: when the therapists’ explained aspects of how the team embraced each other’s reactions, these descriptions were given the tentative code “the team takes care of my feelings and reactions”. To ensure that data material was not left out, HED read through the material that was not coded. HED also commented on KS coding, and the comments were reviewed together. This was done to ensure quality in coding and resulted in some codes being added and others being merged.
3. Keeping our research question in mind, KS and HED searched for potential themes based on our coding. The coding helped us find patterns and clusters of meaning. Relevant codes were sorted under each theme.
4. We then examined closer our tentative themes together with MV. This process helped merge themes together, resulting in three main themes. Data was then reread, with our research question in mind, and presented to ALT and ON to affirm that our themes reflected the participants’ experiences.
5. Themes were then refined, defined and named based on our understanding of what was important in the data set. At this point we presented our themes to the therapist team to gather feedback on whether the themes represented their experiences. Here an important comment on our code “Supervising each other” was made. The participants felt the word supervision represented an uneven balance of power between the receiver and the giver, this did not match their experience with working together. The code was then changed to: “Finding solutions as a team”. This adjustment demonstrates how member checking directly influenced the analytic outcome ensuring the findings resonated with the participants’ lived experience.
6. As a last step, the written thematic structure was agreed upon by all authors, recognizing it as an important story shared by the participants. We summarised therapist experiences into three themes: (a) It can be uncomfortable, but we stick to it; (b) Knowing that your team supports you; and (c) We need to treat people with trauma closer to home.

### Researchers

An important part of qualitative research is to establish how subjective and relational elements may have influenced the research process [39]. At the time of conducting this study, KS and HED were students in clinical psychology and doing their final work placement, ALT was a researcher and clinical psychologist working with trauma patients, ON was a professor in counselling and mental health recovery, and MV was a professor in clinical psychology. Even though we are of different age and gender and are in various stages of our careers as researchers, we are also quite similar. All are born and raised in a wealthy, western society and have well-developed healthcare systems as our main points of reference. During the research process we have reflected and discussed how our own experiences and interests might have influenced our understanding of the data material and how this might reflect in the analysis [31]. At the same time, we have continuously tried to stay as close to the data material as possible in order to bring out the participants’ experiences. This study is reported in accordance with the Consolidated Criteria for Reporting Qualitative Research [40], and is provided in Supplementary File 1.

### Ethical considerations

Weight was put on ethical thoughtfulness throughout the research process. The potential for identifying participants (i.e., the therapists) were discussed with the team at several points. Before taking part, the team members were informed about the project and given the opportunity to ask questions before they gave their written consent. We were also careful not to record data about third persons (i.e., patients).

Although patients were not participants in this project, we wanted to inform them that we were researching the therapist’s experiences with the trauma therapy they were receiving. We also asked for their consent to take part in therapist meetings where information about them would be heard, but not collected. Patients receiving trauma therapy whilst the study was taking place were therefore informed about the project. It was emphasised that participation was voluntary, that their decision would not impact their treatment, and that the focus of this study was not to collect information about them. All patients gave their written consent.

All audio files were deleted after the interviews were transcribed and we omitted personal details from the transcriptions to anonymize them. In accordance with national standards, the project received approval from the Norwegian Agency for Shared Services in Education and Research on March the 6th, 2023.

## Findings

This study aimed to explore therapist experiences when providing intensive-trauma focused treatment as a team, for patients seeking help at Prompt Mental Health Care. We developed three themes that summarised the therapist experiences:

- It can be uncomfortable, but we stick to it.
- Knowing that your team supports you.
- We need to treat people with trauma closer to home.

### It can be uncomfortable, but we stick to it

The therapists explained that the main part of the intensive trauma treatment is to focus on a patient’s trauma. In the focus group interviews the team emphasised the importance of exposure. At the same time, the therapists explained how focusing on a patient’s worst experiences sometimes felt counterintuitive both for the patient and the therapist. We have therefore called this theme *It can be uncomfortable*,* but we stick to it* to express not only the mixed emotions the therapists experienced when focusing on patients’ worst memories in therapy sessions but also their recognition of the importance of sticking to this as the treatment plan. One of the participants referred to this work as exhausting, and noted how it was “intense, both for the people we help, and for us” (Therapist 3). The same therapist, however, noted that trauma therapy that stretched out over weeks and months can be even more exhausting for patients and therapists:

*We*,* as therapists*,* experience that it is difficult for people to come once a week or a couple of times a month for trauma treatment. It can be hard for them to commit to it. When it’s stretched out over weeks and months it can be exhausting for the people concerned. But it can be a bit like that for us too*,* exhausting when it stretches out. Because we have these cases on our own. (Therapist 3)*

In line with trauma therapy being intense, the therapists explained that it could be uncomfortable focusing on patients’ trauma. In the same way as patients found it hard to commit to trauma therapy it seems that the therapists also struggled with similar emotions. One of the therapists explained how she had to find courage to stick to the method.

*You have to trust the method more than you usually do. Sometimes I just have to say this to myself*,* because it is quite counterintuitive when you focus on memories that are so awful*,* and that’s supposed to make sense. Sometimes I have to find the courage and think: “This is where we are going*,* this is the method*,* I have to trust it”. (Therapist 1)*

Another participant explained how he found it difficult to use direct language when speaking about some of the nasty experiences that patients had been through, both with colleagues and with patients. He explained how something in him wanted to skip speaking about, and even imagining, certain experiences. The team then discussed how they had noticed that when practising and learning about trauma-focused therapy, there was a lot of avoidance amongst the therapists taking the course. They explained that during a practice session, therapists took turns selecting trauma scenarios to role-play, embodying the experiences that someone might seek therapy for. They noticed that instead of choosing an assault committed by a family member, therapists tended to choose an accident, a disaster or even being bitten by a dog. The team discussed how they as therapists steered clear of certain images and language to protect themselves:

*Therapist 4: We protect ourselves from that mental image of what it might have been like to be abused by a mother or father. No one wants to imagine that. But still*,* our supervisor plays out these images*,* paints them for us*,* and we can feel it in our stomach. You listen to it*,* and you think “holy shit*,* I can’t quite say that just yet”. So*,* we have to practise to get to that point where we can take it with us into therapy with patients.*

*Therapist 1: You can’t require patients to go there if we don’t.*

*Therapist 4: No*,* that’s absolutely true.*

Another topic the participants focused on in the focus group interviews was how they had previously experienced that focusing on a patient´s trauma could sometimes raise anxiety in them. They explained how they would dread these therapy sessions and were unsure how to treat the trauma in a way that was helpful for the patient. The therapists then went on to discuss earlier thoughts that some patients would not cope with talking about their experiences. One of the therapists then emphasised that she no longer held this view:

*There are a lot of things that are uncomfortable*,* but I don’t associate it with danger or an increased risk of suicide [any more] […] Sometimes people talk about how patients won’t be able to cope with it or that something is going to happen. I no longer believe this. (Therapist 1)*

The same participant focused on how she would have wanted to be treated as a patient herself. She was concerned that by not addressing her patient’s experiences, this might leave them with a sense of having to cope with their trauma alone:

*Therapist 1: The last thing you need is someone that’s scared around you. Who’s insecure and withdrawn and leaves you alone*,* “you’ll have to wait a bit and then we’ll see*,* and then maybe I can”*.

*Therapist 2: “You have to stabilise yourself a bit first”*

*Therapist 1: That’s offering the person loneliness. I don’t want to do that.*

As well as discussing how talking about patient’s worst experience could be counterintuitive, several participants emphasised that they also could experience a feeling of importance and mastery:

*Therapist 2: That’s what’s a bit weird*,* because that’s when you can walk out of a session and just go “YES. It’s happening*,* it’s happening”. Even though it’s been a shitty session.*

*Therapist 3: Yeah*,* that’s exactly it. I notice that I am really focused. It’s something about those feelings that just pull you in*,* it feels so real.*

*Therapist 1: I don’t know*,* just that kind of feeling of managing something well. I can see that the patient is coping with their strong emotions*,* as a therapist I am coping with the strong emotions. There’s a good feeling in it*,* even though it’s painful at the same time.*

The therapists seem to have experienced providing intensive trauma-focused treatment as both being uncomfortable and crucial for recovery. They describe contrasting feelings of importance in the midst of the misery. In sum, this theme captures the therapists’ individual experiences of working with trauma. It reflects the personal strain, ambivalence, felt discomfort and meaningfulness experienced in the therapeutic encounter, before these reactions are shared and contained within the team context.

### Knowing that your team supports you

In the interviews the therapists repeatedly talked about how important working in a team was for them. While the first theme concerns therapists’ personal emotional responses when working with trauma, the second theme illustrates how these individual reactions are received and regulated within the team context. To summarise these experiences, we have chosen to call our second theme *Knowing that your team supports you*. The team explained that when they worked as a team and had several therapist meetings a day, they had a place to react, express and discuss how they felt after focusing on a patient’s traumatic experience. One of the participants said that the team embraced her feelings in a very helpful way. Another therapist emphasised that she did not have to be alone with her experiences:

*When I have patients with strong and intense emotions*,* I don’t have to be alone with it afterwards. I can actually talk to someone. Whereas if I’m in a session with only myself and you’re not connected*,* it’s almost traumatising to have that story told. But here we’re all involved and can embrace each other’s experiences in a very helpful way. So I think that’s an important thing. When you have these intense experiences that affect you*,* to be able to tell someone and be given support. (Therapist 6)*

It was also visible from the observation and field notes that the therapist meetings were a place for the team to focus on their experiences. Here, the therapists could come with their own insecurities and frustrations with an ongoing treatment as well as gratitude and happiness when a patient had a breakthrough. It seemed that the therapist meetings were a place to react as well as to discuss and move on.

When we asked the team in the focus group interviews how they found rotating patients between them, they focused on the value this had, particularly with patients they found difficult to help. When everyone worked together and all therapists in the team had first-hand experiences with every patient, supporting each other became natural and intuitive. Participants also emphasised how useful teamwork was when unsure of the appropriate next step in a person’s treatment or when they did not feel that they were managing the therapy very well. Group discussions and helping each other to adhere to both the treatment plan and the treatment protocol, was also visible when observing the therapist meetings. Moreover, several of the therapists described how valuable it was to be able to discuss the way forward with the team of therapists:

*Therapist 2: Having a team to discuss those difficult cases – “What is [going on]? What do I do? Where should I start?” – and then someone else will take over. That we can look at it as a team… “What is wise to do here? What is it that is making it not move forward?” Because sitting there on your own*,* you’ll be really scratching your head.*

*Therapist 5: You do*,* mhm.*

*Therapist 6: I think it makes you a bit more daring.*

*Therapist 2: Mhm*

Therapist 6: When you meet something difficult

Therapist 1: Yeah, you become a little braver

Another topic that was focused on when addressing therapist rotation was the positive effects of having shared responsibility. One of the therapists put it like this: “It becomes a project that we work on together. Something we do as a team, everyone takes part. We don’t have a patient on our own, right. We find solutions as a team” (Therapist 5). Another therapist responded with: “Yeah. It’s *our* patients.” (Therapist 1). They seemed in agreement that when everyone had a hand in the ongoing therapies, this made them feel more secure in their role as therapists.

*Therapist 1: I think that’s a good point*,* when you have a team behind you that agrees that this is how we’re going to do it*,* then the “doing it”-part is easier to stick with*,* when it gets a bit scary.*

*Therapist 6: Yes*,* when you meet your own kind of blind spots*,* and then you can*,* “no*,* but now [Therapist 3] said or now [Therapist 4] said that you should do this”*,* I have to trust that.*

One of the therapists also focused on how therapeutic challenges were no longer her problem alone. She described how her initial feeling of incompetence could be changed into a puzzle the team had to solve together. They then discussed how their frustrations were addressed and taken care of by the team:

*Therapist 2: And that we kind of own it together as a team*,* it’s not like if I was completely stuck for an hour today*,* that I didn’t manage to change anything*,* that this reflects my incompetence as a therapist in a way. It’s more like “ok*,* now we have a puzzle here*,* can we all get our heads together here. How*,* what should the next person come into this session with to get some movement here?“. It’s something intriguing*,* in a way*,* a shared puzzle.*

*Therapist 1: And I think it has a lot to do with the fact that there are many of us*,* because very often when you come out of a session like that*,* you can come with a lot of frustration*,* “oh my”*,* it’s as if there’s something you’re a bit frustrated about*,* and it’s accepted*,* but we don’t let the frustration become the focus. It’s sort of*,* as you say*,* validated*,* like “hoo” (exhales)*,* then it calms down*,* then we can work on it. And I think that if I’d been alone and frustrated*,* I think it could have rubbed off on some of the continuing work.*

The participants also described that transparency when working as a team also came at a price. On the one side, it kept them on their toes but on the other side it was worrying to have ones work out in the open. One of the participants (Therapist 4) admitted that he sometimes felt uncomfortable when passing on a patient that he had not managed to help particularly well in his previous session. Another acknowledged that it could be quite demanding being so visible to her colleagues:

*We have to dare to be visible to each other*,* what we deliver in therapy sessions*,* that’s always a challenge (…) You have to tolerate that someone watches your films from therapy*,* gives you feedback. So there’s pressure all the time. And then you have to go back and try something new again (Therapist 6)*

Participants described variability in their experiences with the EMDR method, accompanied by collegial encouragement to “dare to trust” the approach. While such encouragement was often intended as supportive, it also generated a subtle yet persistent sense of performance pressure. The rotational structure of the team was experienced as reassuring insofar as responsibility for the case was shared and another colleague would assume clinical care. At the same time, this model required therapists to make their clinical work continuously visible to others. Transferring a client following a session perceived as unsuccessful necessitated openly acknowledging one’s own doubts or perceived shortcomings. Although participants emphasised the professional benefits of transparency, such as shared learning, collective responsibility, and strengthened clinical quality, the rotational arrangement also entailed an ongoing preparedness to have one’s clinical judgments and decisions scrutinized by colleagues.

The therapist team focused on the benefits when working as a team whilst providing trauma-focused treatment. They explained that as therapists they felt supported and taken care of by each other. Whilst the therapist meetings provided an opportunity to express their own reactions, it also meant that they were visible to their colleagues.

### We need to treat people with trauma closer to home

In the interviews, the participants emphasised how trauma is a common issue and stressed that evidence-based treatment for trauma must be provided at a lower level of care. We have called the third theme “We need to treat people with trauma closer to home” to express the therapists’ view that trauma-focused therapy should be available in their local community.

The therapist team challenged the traditional understanding of trauma as phenomena reserved for treatment at a higher, more specialised level of care, because this risked services being unavailable for many people who might need it. One of the therapists questioned the way trauma therapy was delivered today: “One doesn’t have to think of it as something completely specialised, that you have to become very, very ill before you’re allowed access to it” (Therapist 1). Similarly, another participant highlighted how frequently trauma is in the lives of people with various mental health problems: “The underlying reason for anxiety and depression is often trauma. That’s why we should focus on where the problem started and not work with the symptoms that are visible on the surface” (Therapist 6).

This first therapist also emphasised that working with people’s traumatic experiences was neither very difficult nor took a lot of time. She elaborated:

*Single-incident traumas are easy to treat with EMDR. That’s why I think they should be treated at the Community Mental Health Services instead of sending them on to the specialist mental health care system. It’s often short-term treatment. (Therapist 1)*

The health centre’s focus on trauma was at the outset established as a response to a helicopter accident in the local community. Multiple fatalities and many people witnessing this event called for a rapid development of services. All therapists were educated in EMDR as a way of helping people that were suffering because of the accident. Subsequently, participants felt there was a need to provide trauma-focused treatment in the community as a supplement to existing treatment services, in order to make such interventions available to a broader group of individuals experiencing trauma-related symptoms.

Both in the interviews as well as in the observations therapists reported a strong motivation to initiate treatment early, as they perceived this as potentially reducing patients’ vulnerability to further traumatic experiences. The same participant as quoted above, put it like this in one of the therapist meetings that we observed: "If she had been able to process her traumas earlier, the ones from childhood, you can ask yourself whether she might not have had to experience this in adulthood. Then it might not have happened again" (Therapist 1).

Another important aspect of this theme was the therapists’ experiences with providing trauma therapy digitally. The therapists felt that the service being delivered digitally lowered the threshold for patients taking part and through this medium they reached people who otherwise would not have attended a clinic. The digital format also made it possible for people who could not receive therapy twice a day, because of the long distance between the health care centre and their homes.

The therapist’s team were in broad agreement that intensive trauma therapy provided as a team was easier to provide online. However, the participants believed that other therapists were sceptical to treatment online, especially trauma treatment. They discussed how the idea that one must take care of patients’ feelings, might stand in the way of therapists using a digital format for trauma therapy:

*Therapist 6: how much are you as a therapist responsible for the feelings your patients have? It is possible to think that your patient is able to deal with their feelings themselves. I don’t have to think that I should be the one to deal with them. I facilitate the processing part*,* but the patient is trained to deal with their feelings all the time themselves. So*,* I think that’s why*,* that you think that “if I’m there*,* I can sort of take care of them [the feelings] for you”. Whereas here they’re a bit further away and they’re left on their own.*

*Therapist 2: And that’s just rubbish. When someone comes to your office as an outpatient*,* you don’t take more care of what happens between appointments.*

To summarise, the team of therapists seemed in agreement that trauma therapy should be provided at the lowest level of care to prevent people going untreated. They also shared the view that a digital format made the therapy easier to provide and lowered the threshold for patients to access and engage in treatment.

## Discussion

In this study we explore how therapists experience providing intensive, team-based trauma-focused treatment for patients seeking help at Prompt Mental Healthcare. We summarised these experiences into three themes (a) *It can be uncomfortable*,* but we stick to it;"* (b) *Knowing that your team supports you;”* and (c) *We need to treat people with trauma closer to home.*” In the following we discuss our findings and how they contribute to existing knowledge.

### Teamwork in the face of trauma: exploring collaborative approaches

Previous research has identified therapist fear and discomfort as key barriers to the delivery of trauma-focused treatment [22–24, 45]. In addition, studies have shown that healthcare professionals working with trauma have higher rates of secondary traumatic stress and burnout [41–44]. This can explain why TFTs are underused despite the evidence of their effectiveness for people with post-traumatic stress disorder [8, 21]. Our findings extend this literature by illustrating how therapist rotation may change how such fear is managed in practice.

The observation that traumatic memories evoke discomfort in therapists [23–24, 45] is consistent with findings observed in our first theme, “It can be uncomfortable, but we stick to it”. Our findings suggest that the therapists tolerate their own discomfort because they trust the efficacy of the intensive format. This aligns with recent research on patients’ experiences with a similar intensive trauma treatment. Vaage-Kowalzik et al. found that most of the patients experienced the intensity of the treatment program as necessary to stick to the processing of their trauma history [46]. At the same time patients also reported that there were times where they felt too overwhelmed to benefit from parts of the treatment [46]. These patients’ perspectives serves to validate the therapist’s intuition in our study: that ‘sticking to it’ through the discomfort is essential for the patients’ long-term recovery.

Collective containment can be understood as an organizationally distributed form of the psychoanalytic function Bion called containment: transforming overwhelming, unprocessed affect into something thinkable within a relational context [47]. In trauma work, this task exposes clinicians to intense material that can exceed individual coping resources. Potic notes that PTSD treatment may require therapists to contain “destructive” material that can harm them emotionally, raising the question of who contains the therapist [48].

Our findings suggest that therapist rotation operates as a collective containment mechanism: the emotional and moral burden of traumatic work is shared across the team rather than privately carried by one clinician. This may reduce cumulative strain associated with vicarious trauma and buffer moral distress by making difficult experiences discussable, comparable, and co-owned through a structured routine. In this sense, collective containment is not only interpersonal support, but a form of organizational emotion regulation that helps sustain clinicians’ capacity for empathic engagement and safe, consistent care.

In our study, therapists managed their discomfort, fears, and reactions by collaborating as a team. This team approach is particularly noteworthy in summarizing and understanding the therapists’ experiences. Specifically, the shift from an individual responsibility (“I, as a therapist”) to a collective approach (“We, as a team”) is significant. While clinicians typically bear individual responsibility for their patients, our second theme, “Knowing that your team supports you,” highlights how the emphasis on teamwork in this context proved both functional and emotionally supportive for the clinicians. Individual responsibility risks placing a larger burden on therapists as they have to cope on their own not only with their own reactions, but also with challenges within treatment. Working in a team with meetings between TFT sessions seems to be a solution to lessen the burden for therapists and creates a space to share difficulties and reactions.

For us as researchers it seems that the team of therapists created a climate that was high on trust and psychological safety. Edmondson [49–50] explains psychological safety as a shared belief that it is safe to take interpersonal risk. This safety enables team members to confidently speak up and ask for help because there is a reduced risk of embarrassment and increased trust in each other. Hunt et al. [51] emphasizes the critical role of psychological safety in mental health services, as effective decision-making requires professionals who are able to voice their perspectives. Kroon et al. [52] qualitative study also found that trust and transparency in relationships was looked upon as an important aspect of collaboration in a mental health care system in the Netherlands, however this was often experienced to varying degrees. We believe that prioritizing psychological safety, alongside mutual trust amongst team members will positively improve teamwork.

A commonly reported barrier to providing TFT amongst therapists is the concern that patients’ symptoms may worsen or that they might experience retraumatization [22]. Finch et al. [22] discusses how the minority that have an increase in symptoms during treatment are still highly likely to experience post-treatment clinically significant symptom improvement. A Norwegian implementation study of evidence-based treatment (EBT) for PTSD [53] showed that EBT was effective both for patients with PTSD and for patients with probable Complex PTSD. Furthermore, only a few patients reported an increase in symptoms during treatment. The therapists in our study also reported that they had a fear that patients wouldn’t cope with a trauma focus, however this changed having learned EMDR and providing it in a team. Van Minnen et al. [20] suggested that when therapists provide TFTs together, therapists can safely learn by experience that their fears, for example of patients not coping with a trauma focus, are not realistic. Our observations support this. Whilst observing the therapist meetings it was visible that the team supported each other in adhering to both the treatment plan and treatment protocol. Our study therefore expands previous findings as it shows how working in a team has the potential to reduce therapist drift, which is the phenomenon of clinicians drifting away from the treatment protocol or choosing not to use EBTs [54]. Our findings resonate with Vaage-Kowalzik et al., who also reported that rotation-based teamwork reduced therapists’ fear and supported protocol adherence in intensive trauma treatment [25]. While their study focused on an inpatient context, our results show that similar collaborative approaches can be mobilised in primary-level, digitally delivered services. This suggests that rotation-based models may have wider relevance across organizational levels, particularly when combined with structures that foster psychological safety and shared responsibility. We believe that working in a team reduces barriers and implementation difficulties associated with providing evidence-based therapy. This collaborative approach could increase access to evidence-based treatments, a topic we explore in our third theme: “We need to treat people with trauma closer to home.”

Our findings suggest that therapist rotation within a well-functioning team may yield important benefits, including shared responsibility, mutual emotional support and collective learning. Previous studies of multidisciplinary teams in paediatric oncology [55] and in services for individuals with severe and persistent mental disorders [56] similarly report that opportunities for debriefing and access to colleagues’ expertise are experienced as protective and clinically valuable. While these studies concern teamwork more generally rather than therapist rotation specifically, the parallels suggest that structured collegial support plays a crucial role in managing emotionally demanding work.

However, our findings also indicate that rotation introduces particular demands. Working in a transparent rotation system requires therapists to make their clinical decisions visible, remain open to feedback and tolerate performance pressure. Although participants emphasised the benefits of shared responsibility, they also described the personal cost of exposing perceived shortcomings. It is therefore reasonable to assume that teams characterised by reduced trust and psychological safety may not experience the same gains. In less cohesive contexts, such transparency could intensify anxiety or defensiveness rather than facilitate learning and emotional regulation.

### Implications

This exploratory study may have important implications for mental health care practice. Firstly, as highlighted in our first theme, therapy with people that suffer the effects of trauma can be painful and requires mental health professionals that dare explore the horrible experiences people have lived through. These findings suggest that mental health systems could benefit from integrating mechanisms that prevent staff burnout and increase staff wellness. Secondly, as described in our second theme, our findings highlight the potential value for therapists and healthcare leaders in adopting a team-based approach when working with trauma patients. In our study, the trust and support within the team fostered courage and confidence that made it easier to stick to a treatment that was challenging both for the patient and therapist. While these team processes are often described as peer supervision, our findings suggest a conceptual move towards collective containment. Following Moss [57], this differs from standard supervision by focusing less on retrospective skill-building and more on the active, real-time processing of traumatic material shared across the team. From an implementation perspective, such collective containment can be understood as an adherence mechanism: by distributing and processing emotional burden at the team level, it reduces pressure on individual therapists and makes it easier to stay with the intensive trauma-focused protocol rather than drifting away from it. Another central implication of our finding is the importance of a team context in facilitating therapists’ self-care. The team context with regular team meetings provides a space where therapists can seek emotional support from each other. The team setting might also make it easier for therapists to set healthy boundaries between work and personal life by discussing and debriefing complex cases that one otherwise “takes home”. Our analysis expands upon previous findings showing that working in a team, with therapist rotation, can protect therapists from the effects of work stress by promoting a space where therapists could take care of themselves and each other. The benefit of working in a team might also have important implications for those early in their careers or in lower status positions, as these groups might experience low levels of psychological safety [58]. Thirdly, as described in the third theme, providing trauma-focused treatment close to where people live, offers the opportunity to provide people with low threshold level treatment. Digital innovations may offer a possible avenue for addressing implementation challenges as suggested by Vigo [59], as such innovations have significantly altered the opportunities within mental health policy and systems [50]. Our study shows that online treatment can feel as safe to provide as non-online treatment. Our findings illustrate how digital formats can support more equitable access to care to populations in rural areas. This meets the challenges of long distances, which is the case in many small communities in Norway. This study has only focused on therapists’ experience with providing intensive team-based trauma-focused treatment for patients accessing services at Prompt Mental Health Care. As we did not interview patients, who have the most important experiences of treatment and care, their experiences of this treatment need to be explored. To develop useful and meaningful teamwork in the mental health field future studies should therefore focus on how patients experience team-based therapy.

### Reflexivity

Reflexivity involves researchers’ thoughtful analysis of their own position in every phase of the research process [39], what Braun et al. [32] describe as “the process of a researcher critically reflecting on their values, assumptions, expectations, choices and actions throughout the research process and considering what these might enable, exclude and close off” (p. 22). Therefore, emphasising reflexivity creates a dual focus in research studies as both the phenomena of investigation as well as the process of conducting research become aims of attention [60]. It requires researchers to take two steps back: The first is from the observation of the research and the second is from the reflection of the observation itself [61].

In conducting the present study, the position of the principal researcher (KS) can be seen as fluctuating between that of an insider and that of an outsider [62]. KS having undertaken a final work placement at the research site was an insider in the sense of being familiar with the context, the participants, and the treatment they provided. This enabled a privileged position of trust, which was important in the process of collecting data through observations and interviews. It contributed to a willingness of participants to open their doors to their offices, include KS in their ongoing discussions and share their experiences in the interviews. This assisted the development of a valuable and rich dataset in the present study. At the same time, KS held the position of an outsider. As a student in clinical psychology, there was an imbalance of power between KS and the group of professionals. In this we could sense a risk of becoming strained and inhibited. The ambiguity of this dual position, being simultaneously an insider and an outsider, was both challenging and beneficial. To explore this uncertainty of being in-between and to ensure analytic trustworthiness, frequent meetings were conducted with the second author (HED) with supervision provided by the last author (MV). This allowed us to balance the first author’s insider closeness with the co-authors’ critical distance. Here we addressed the experience of being somewhere in the middle – not yet a therapist and not yet a researcher. This process helped us recognize how being in flux also creates a dynamic, multi-faceted research lens, allowing us, for example, to be sensitive to the research context and to ask questions from an unknowing stance. We believe this position enabled stories to be told and for the interviewers to attune to them in a responsive way.

As a research team we may have influenced the research process in several ways. We have lived experiences of traumatic events happening to ourselves or someone close to us, which may have impacted our engagement with the field and helped us acknowledge how common traumatic experiences are. It is something that truly can happen to us all. However, to maintain analytic trustworthiness, we actively reflected on these personal resonances during the analysis to ensure that our interpretations, particularly regarding the emotional burden of the work, were firmly grounded in the participants’ accounts rather than our own professional and personal experiences. Furthermore, although we are of different genders, from various parts of Norway, and find ourselves at diverse points in our careers as researchers and therapists, we are probably more alike than different. Consequently, we may have overlooked perspectives on trauma work that are at variance with the more conventional approaches in our mental health system. For example, although we recognize that there is a substantial diversity in what people consider traumatic, our background and experiences may have made us blind to the more continuous trauma that may follow structural racism and other kinds of systemic oppression, and perspectives on how this needs to be addressed in the role as a therapist.

### Study strengths and weaknesses

Reflexive thematic analysis is considered a useful approach to articulate and explore therapists’ lived experiences [63–64]. Our extended observation of therapist meetings and the use of multistage focus group interviews made it possible to learn over time about their experiences of working together to provide TFT for patients seeking services from Prompt Mental Health Care. We are, however, aware that a deeper exploration of the therapists’ experiences could have been engaged if we had interviewed participants individually and in-depth. In particular, perspectives that are at odds with other therapists’ views could have come to light more easily in a one-to-one setting. Although our analysis includes accounts of performance pressure and the demands of openly sharing one’s clinical work, these aspects were not explored in the same depth as more supportive and affirmative experiences. This may partly reflect a positive bias associated with studying a well-functioning team, where strengths and adaptive processes were more readily articulated. It is also possible that more critical, vulnerable or dissenting perspectives might have emerged more fully had we conducted individual in-depth interviews in addition to focus groups. As argued, we consider our data material as rich, and we believe that our research carries methodological integrity through systematically basing our findings in the field notes and transcribed interviews. However, given the complex nature of the topic, it is unlikely that we explored the full range of interactions between therapists and the depth of their experiences. Future research could therefore usefully consider expanding upon our study findings as well as exploring therapists’ experiences with different approaches to trauma work. For example, although the treatment in this study was delivered online, therapists’ experiences related specifically to the digital format were less prominent in the data than anticipated. Future studies could therefore examine more explicitly how digital delivery shapes therapists’ experiences, therapeutic processes, and team-based work in intensive trauma-focused treatments. Furthermore, our study describes the value of working together towards helping people heal their traumas. This may be because we explored a well-functioning team, and supplementary interview studies could contribute to further knowledge about the challenges related to working as a team in the Community Mental Health Services. Owing to our rural research context, all participants were ethnic Norwegians. Accordingly, there was clearly a lack of cultural diversity in our sample. The transferability of our findings may therefore be limited by this specific sociocultural and organizational context, characterized by high institutional trust and a traditionally egalitarian work culture. Such factors may have uniquely facilitated the implementation of therapist rotation and team-based trauma work. Readers should be mindful of this limitation and care needs to be taken when transferring study findings to other contexts.

## Conclusion

Our findings reflect therapists’ experiences of providing trauma treatment for patients accessing services at Prompt Mental Health Care. We report three themes that describe their perspectives: (a) It can be uncomfortable, but we stick to it; (b) Knowing that your team supports you; and (c) We need to treat people with trauma closer to home. This study has revealed that working in a functional team, within a framework of therapist rotation, can make it more feasible for therapists to provide TFTs due to the shared responsibility and support within the team. Our analysis also expands on previous findings showing that teamwork can protect from the adverse effect of work stress by promoting a space where therapists can take care of themselves and each other. The insights gained from our study can have implications for the organisation of future healthcare systems, leading to improvements in access to evidence-based treatments and promoting better working conditions for therapists. Future qualitative and quantitative research should investigate the specific team characteristics and dynamics that enable therapist rotation to be beneficial rather than destructive. Understanding what makes a team ‘functional’ in terms of communication patterns, leadership, shared values, and mutual respect, would be crucial for wider implementation of this model in other settings and services.

## Supplementary Information

Below is the link to the electronic supplementary material.

Supplementary Material 1

Supplementary Material 2

## Data Availability

Due to the privacy of the participants the dataset analysed during this study is not publicly available. The dataset can be made available on reasonable request from the corresponding author.

## Abbreviations

TFT: Trauma- focused treatment
TE: Traumatic event
PTSD: Post-traumatic stress disorder
TF-CBFT: Trauma-focused cognitive behavioural therapy
EMDR: Eye Movement Desensitization and Reprocessing
iEMDR: Digital Eye Movement Desensitization and Reprocessing
PE: Prolonged Exposure
KS: Kristin Stotesbury
HED: Hilde Einarsdatter Danielsen
MV: Marius Veseth
ALT: Anders Lillevik Thorsen
ON: Ottar Ness
EBT: Evidence-based treatment
