# Supplementary material for: Confronting trauma as a team: therapists’ experience with providing intensive trauma-focused treatment, within a framework of therapist rotation
Source: BMC Health Serv Res. 2026 Apr 14;26:744. doi: 10.1186/s12913-026-14497-z (PMC13202738; doi:10.1186/s12913-026-14497-z)
Supplement: Supplementary file 1 — Supplementary Material 1 [file 12913_2026_14497_MOESM1_ESM.docx]

**Focus group 1 – Interview guide**

**1.0 Community Mental Health**

1.1 What is the reason you want to work with intensive trauma treatment in the Community mental health service?

1.2 What do you want trauma treatment in the community/municipality to be, in contrast to what is offered in the specialist health service?

**2.0 Intensive EMDR over the Internet**

2.1 What is it about this form of treatment that you find effective?

- Do you have examples of this?

2.2 What challenges do you experience with this form of treatment?

- Do you have examples of this?

2.3 Has intensive EMDR made you think differently about trauma treatment?

**3.0 Team**

3.1 What is it like to work in a team with intensive EMDR?

3.2 How do you perceive the role of the team

- Do you have examples?

3.3 What do you value about working in a team when doing intensive EMDR?

- Do you have examples?

3.4 Do you have examples where it has been challenging to work in a team?

**4.0 Therapist Rotation**

4.1 How do you find “therapist rotation” during intensive EMDR?

- Do you have any examples where therapist rotation has been beneficial?
- Do you have any examples where therapist rotation has been challenging?

**5.0 Innovation**

5.1 Is there anything you wish you could change about the current treatment approach?

5.2 Is there anything we haven’t asked about that you would like to add?
