# Supplementary material for: Confronting trauma as a team: therapists’ experience with providing intensive trauma-focused treatment, within a framework of therapist rotation
Source: BMC Health Serv Res. 2026 Apr 14;26:744. doi: 10.1186/s12913-026-14497-z (PMC13202738; doi:10.1186/s12913-026-14497-z)
Supplement: Supplementary file 2 — Supplementary Material 2 [file 12913_2026_14497_MOESM2_ESM.docx]

**Focus Group 2 – Interview guide**

**1.0 Sticking to the Method**

**Quote from the focus group interview:**

*Therapist 6: “I don’t think we should freestyle to much, I don’t think we should let the patients choose.”
Therapist 3: “At least not when they’re good at beating around the bush.”
Therapist 2: “... I think we need to steer. Not talk. Go straight to the targets.”
Therapist 4: “Yes, it’s a difficult group.”
Therapist 1: “We need to be strict with three out of four. Because they’re good at diverting us to something else.”
Therapist 2: “I get led into it (the conversation). We need to be good at staying true to the method.”*

**Question:** If I were to summarize what I noticed during the observation, it was how you helped each other stick to the method. Do you agree with that? Is there anything you would like to say about it?

Here is another situation where I felt you were discussing the method:

*Therapist 1: “So, she needs to be talked to occasionally.”
Therapist 3: “Don’t talk too much. We know you like that.”
(Lots of laughter)
Therapist 1: “What if I do…?”
Therapist 3: “As long as you do EMDR, V.V. As long as it’s EMDR.”
Therapist 1: “Why can’t we focus on those memories (the patient’s request)?”
Therapist 3: “Because the SUD (Subjective Units of Disturbance) is lower.”
Therapist 1: “But this is what she’s bringing up. She wants to focus on this and that, and then we bring something else.”*

**Question:** What are your thoughts when you heard this?

**2.0 It’s Emotionally Intense to Work with Trauma**

**Quote from focus group interview 1:**

*“It can be quite intense to be with people who muster up the courage to look straight into things they have done so much to avoid for many, many years. So, I think it’s quite powerful experiences.”*

*“You have to trust the method more than you usually do. Sometimes I just have to say this to myself, because it is quite counterintuitive when you focus on memories that are so awful, and that’s supposed to make sense. Sometimes I have to find the courage and think: “This is where we are going, this is the method, I have to trust it”.*

**Questions:**

- What do you do to manage this intensity?
- What is it like to work with something so emotionally intense over the internet?
- How do you feel after a session like this?
- What do you do to get back on track/reground yourselves?
- (What do you do as a team to get back on track/reground yourselves?)

**3.0 Trauma Treatment is Not as Uncomfortable Anymore, Why?**

To summarize how intensive EMDR has changed your thinking about trauma therapy, I’d like to read some quotes from the previous interview:

“*People can actually tolerate trauma treatment, not just once a week, but intensively, twice a day for an entire week.” (*Quote from focus group interview 1)

You want to challenge the myth that everything has to be so stable.

*“There are a lot of things that are uncomfortable, but I don’t associate it with danger or an increased risk of suicide [any more] […] Sometimes people talk about how patients won’t be able to cope with it or that something is going to happen. I no longer believe this.” (*Quote from focus group interview 1)

It’s as if you’re saying that the way we’ve structured this—with therapist rotation, a team, and supervision between each session—makes it safer for you as therapists to do trauma treatment.

**Questions:**

- Have we understood this correctly?
- What is it that makes it feel safer when you operate this way?
- Is there anything more you’d like to add?

**4.0 It’s Fun, Why?**

**Quote from the previous interview:**

*Therapist 1: “Yes, I think it’s a demanding week.”
Therapist 6: “Mhm, yes.”
Therapist 1: “But like, it’s also a crazy mix of having a lot of energy in the room that week. So, I want to go to work, it’s very much like that.”
Therapist 6: “I find it very empowering for the team feeling, yes.”*

You’ve mentioned that during the week of EMDR, there’s “*a lot of energy in the room”* Many might think, when they hear about intensive EMDR over the internet, and on top of that, therapist rotation, “That’s definitely not something I want to be involved in.”

**Questions:**

- Do you have anything you’d like to say about this?
- What is it that creates so much energy in the room?

**5.0 Challenges with Intensive EMDR**

**Quote from the participatory observation:**

*Therapist 3: “I think we’ll manage with her.”
Therapist 2: “Yes, yesterday I felt very uncertain.”
Therapist 3: “I think it’s very similar to previous patients. And there I thought, this won’t be easy.”
Therapist 2: “Yes, I felt I didn’t move her an inch.”
Therapist 1: “Yes, that’s not easy to sit with.”
Therapist 2: “No, it’s not easy at all. I felt, ugh.” (sighs)
Therapist 1: “We have to hold on to the fact that we’ve had patients that go up and down. That progress goes up and down. It stops sometimes.”
Therapist 2: “I wouldn’t have been able to sit with her alone. It’s really good that someone else will take over.”*

**Questions:**

- What do you think when you hear this?
- *“I wouldn’t have been able to sit with her alone*” Can you say something about what it’s like to be part of a team in this context?

**6.0 Therapist Rotation**

If I try to summarize what you’ve said about therapist rotation, you mention that it can sometimes be challenging to hand over a patient, because you have to somehow show what you’ve accomplished in the session, but other times it’s a relief that someone else is taking over.

**Questions:**

- Does this summary reflect your experience? Have we understood you correctly?
- Tell us more about this!
- Do you have a specific example of...
- Tell us about a time when therapist rotation worked really well.
- Tell us about a time when therapist rotation was very challenging.
- How is it to have a new patient for each session?

**If time:**

**7.0 The Relief of Having a Method to Use**

**Quote from the interview:**

*“When I worked at DPS (District Psychiatric Center) before, I felt that all those cases were kind of, they perhaps triggered some anxiety in me because I didn’t really know how to handle them or approach them (...) There was a focus on symptom reduction instead of processing the root of the traumas, and it hasn’t been nearly as effective as EMDR.”*

**Question:**

- Am I correct in understanding that there’s a kind of relief in finally having a tool that works? Can you say more about this?

**8.0 Shared Responsibility for Treatment**

**Quote:**

*“I think it’s really, it’s really nice to have that sense of community. It becomes very much ‘our patients,’ (...) I can feel that if I’ve done the screening or first conversation with a patient, suddenly I have five others who are just working and cheering for my patient. And then it’s not mine anymore, it’s our patient. There’s something incredibly, yes, powerful about that.”*

**Question:**

- Would you like to say more about this?
